# Successful management of free osteocutaneous fibula flap with anomalous vascularity of the skin paddle

**DOI:** 10.4103/0970-0358.59295

**Published:** 2009

**Authors:** Prabha S. Yadav, Quazi G. Ahmad, Vinay Kant Shankhdhar, G. I. Nambi

**Affiliations:** Plastic and Reconstructive Services, Department of Surgical Oncology, TATA Memorial Hospital, Parel, Mumbai – 400 012, India

**Keywords:** Anomalous perforator, double vascular anastomosis, osteocutaneous fibula flap

## Abstract

The free osteocutaneous fibula flap is an established method of reconstruction of maxillary and mandibular defects. The vascularity of the skeletal and the cutaneous components is provided by the peroneal artery via the nutrient artery and the septo- and musculocutaneous perforators. In rare situations, these perforators may arise from other major leg arteries. In such circumstances, the procedure has to be either abandoned or modified so that neither the vascularity of the flap nor the donor limb is compromised. We present a case of an anomalous musculocutaneous perforator, which originated from the proximal part of the posterior tibial artery, passed through the soleus muscle and supplied the skin paddle. The flap was elevated as a single composite unit and was managed by two separate vascular anastomosis at the recipient site, one for the peroneal vessels and the other for the anomalous perforator.

## INTRODUCTION

The reconstruction of oro-mandibular defect after tumour ablative surgery is a challenging task in terms of anatomical and functional results and this has been fulfilled by the free fibula flap. Ever since described, the free fibula flap has become the procedure of choice for treating post tumour excision of oro-mandibular defects in majority of the cancer centers. Over the years, there are literature reports of various modifications in the surgical technique and so do the reports of variations of normal anatomy of the leg and that of the flap. We describe a unique situation involving the perforators supplying the skin paddle of the flap which is significant in terms of flap survival and management.

## CASE REPORT

**Figure 1:**
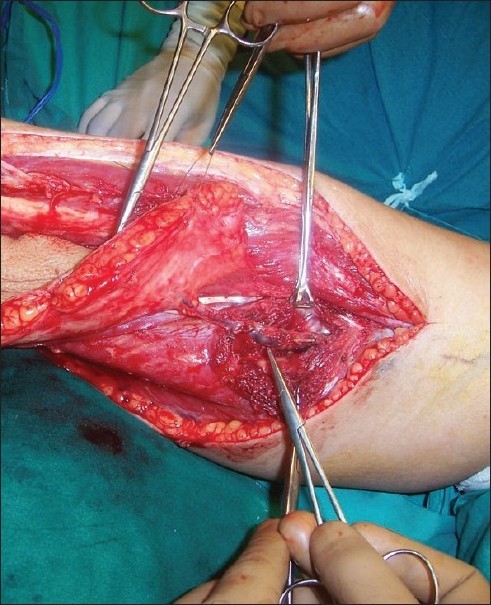
The anomalous musculocutaneous perforator being dissected through the substance of the soleus muscle

**Figure 2:**
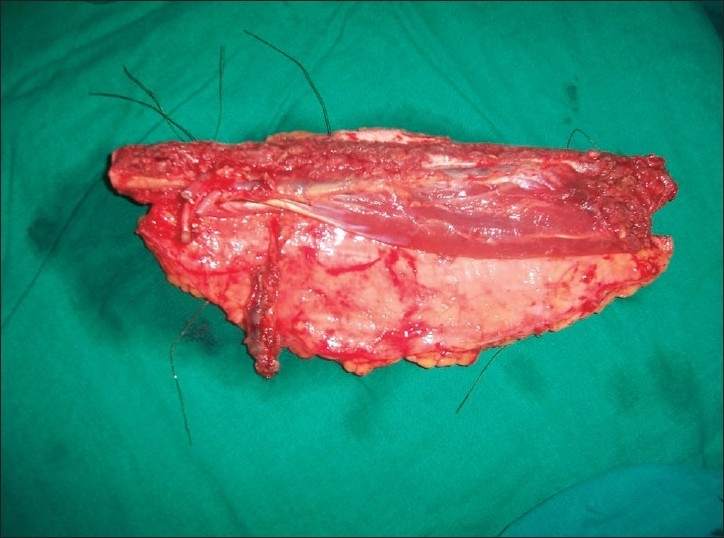
The free fibula flap with the peroneal vessels and the anomalous vessel supplying the skin paddle

**Figure 3:**
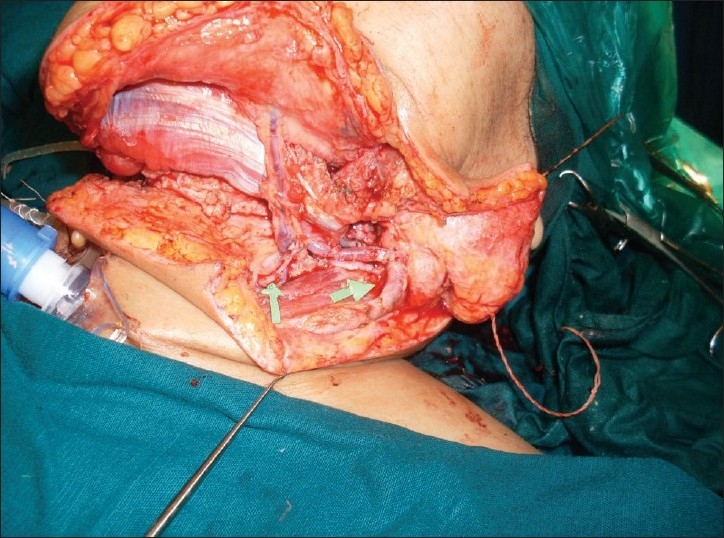
The medial arrow depicting the anastomosis of the anomalous perforator and its venae commitantes and the lateral arrow depicting the anastomosis of the peroneal vessels

A 44-year-old female who was diagnosed with carcinoma of the lower alveolus with involvement of the middle third of the mandible, anterior floor of the mouth, gingivo-labial sulcus and the overlying skin. It was planned for composite resection followed by reconstruction with free fibula osteocutaneous flap. During the preoperative assessment, her dorsalis pedis and posterior tibial pulsations in both legs were normal by palpation. The left leg was selected and the fibula with a skin paddle of 22 × 9 cm was raised through standard anterior approach under tourniquet control. After the distal and proximal osteotomies and after ligating the distal end of the peroneal vessels and moving proximally, no septocutaneous perforators were noticed. Further dissection revealed a single musculo cutaneous perforator coming out of the soleus muscle and proceeding to the skin paddle. With the possibility of anomalous perforator in mind, the vessel was dissected along its entire length through the substance of the soleus muscle [Figure 1] and was found to be originating from the posterior tibial artery 2 cm below the trifurcation. In order to be doubly sure about the contribution of this anomalous vessel to the skin paddle, we applied microvascular clamp to this vessel after releasing the tourniquet. To our surprise there was no bleeding from the margins of the skin paddle and bleeding was restored after the release of the clamp from the anomalous vessel confirming that this was the only vascular supply to the skin paddle. The rest of our flap dissection went normally and the flap was harvested as a single composite unit with two vascular pedicles [Figure 2] and the donor site was closed partly with split-skin graft. After the necessary osteotomies were completed in the flap, the bone fixation was done and the intraoral inset of the flap was given. The peroneal vessels were anastomosed to the left facial artery and the left external Jugular vein and then the bleeding from the skin edge was noted once again and was found to be nil. The skin margin bleeding was satisfactory when the anomalous vessel and its and its venae commitantes were anastomosed to the left superior thyroid artery and a tributary of the left internal jugular vein [Figure 3]. After confirming good bleeding from the margins of the skin paddle, the final inset of the flap measuring 19 × 8 cm was given covering the floor of the mouth, inner and outer aspects of the lower lip and the chin. There were no postoperative complications and the flap settled well and the patient was referred for adjuvant radiotherapy.

## DISCUSSION

The osteocutaneous fibula flap was described by Taylor[1] and later Hidalgo[2] reported its first application in mandibular reconstruction. Since then, it has remained the method of choice of mandibular reconstruction after trauma and tumour-ablative surgeries. The vascular supply to the skin paddle of the flap is through the septocutaneous perforators[3] arising from the peroneal vessels and the standard method is to place the skin paddle in the lower leg.[4] These perforators are consistently present though they may vary in number and very rarely may be absent or arise from lower limb arteries other than the peroneal artery.[5,6] When the septocutaneous perforators are deficient or absent, the vascular supply of the skin paddle is from the soleus musculocutaneous perforators. Like their septocutaneous counterparts, the musculocutaneous perforators can arise from any of the leg vessels, though the majority [50%] of them arises from the peroneal vessels. The rest of them arise from the posterior tibial vessels or from the trifurcation.[7] Depending upon the source of origin, the soleus musculocutaneous perforators can be convergent or divergent.[7] Convergent perforators are those which arise from the peroneal vessels and have the advantage of being harvested as a single composite unit along with the flap. Divergent perforators are those which arise from vessels other than the peroneal vessels i.e.posterior tibial or trifurcation, and therefore, the skin and the bone components have to be harvested separately and require two separate vascular anastomosis at the recipient site. In such situations, the alternative options are:

- Remove the skin paddle from the flap[6] or
- Use fibula for bony reconstruction and another flap such as the radial forearm flap for skin and soft tissue[8] or
- Abandon the procedure and use the other limb.

In our case, we were prudent enough to dissect and include the anomalous perforator after confirming that it was the only source of blood supply to the skin paddle. Though there are reports of anomalies involving the axial arteries of the leg[9] and definite contraindications for the harvest of fibula osteocutaneous flap[9] there are only a few reports of anomalies involving the perforators.[5–7] There are reports of successful management of fibula osteocutaneous flaps with anomalous vascular supply of the skin paddle,[10,11] but ours is a unique case in that a flap with a divergent type of soleus musculocutaneous perforator was harvested as a single composite unit and was managed successfully with two separate anastomosis at the recipient site with no complications of the donor or recipient site.

To conclude, the anomalies involving the perforators may be minor when compared to the major leg vessels but awareness about the possibilities will enable the surgeon to salvage the free fibula osteocutaneous flap as a single unit or as two separate [skin and bone] units with appropriate number of anastomosis as required for successful reconstruction.
